# Investigating the Relationship Between Physical Activity Disparities and Health-Related Quality of Life Among Black People With Knee Osteoarthritis

**DOI:** 10.5888/pcd20.220382

**Published:** 2023-07-06

**Authors:** Donya Nemati, NiCole Keith, Navin Kaushal

**Affiliations:** 1 Department of Health Sciences, School of Health and Human Sciences, Indiana University, Indianapolis, Indiana; 2 Department of Kinesiology, School of Health and Human Sciences, Indiana University, Indianapolis, Indiana

## Abstract

**Introduction:**

Knee osteoarthritis (OA) is the most common form of arthritis, which is a leading cause of disability. Although no cure exists for knee OA, physical activity has been shown to improve functionality, which can improve an individual’s health-related quality of life (HR-QOL). However, racial disparities exist in participating in physical activity, which can result in Black people with knee OA experiencing lower HR-QOL compared with their White counterparts. The purpose of this study was to investigate disparities of physical activity and related determinants, specifically pain and depression, and how these constructs explain why Black people with knee OA experience low HR-QOL.

**Methods:**

Data were from the Osteoarthritis Initiative, a multicenter longitudinal study that collected data from people with knee OA. The study used a serial mediation model to test whether a change in scores for pain, depression, and physical activity over 96 months mediated the effects between race and HR-QOL.

**Results:**

Analysis of variance models found Black race to be associated with high pain, depression, and lower physical activity and HR-QOL at baseline and month 96. The findings supported the prospective multi-mediation model, which found pain, depression, and physical activity to mediate between race and HR-QOL (β = −0.11, SE = 0.047; 95% CI, −0.203 to −0.016).

**Conclusion:**

Disparities in pain, depression, and physical activity could explain why Black people with knee OA experience lower HR-QOL compared with their White counterparts. Future interventions should address sources of pain and depression disparities by improving health care delivery. Additionally, designing race- and culture-appropriate community physical activity programs would help to achieve physical activity equity.

**Summary**

**What is already known on this topic?**

Knee osteoarthritis is a debilitating condition that compromises a person’s health-related quality of life.

**What is added by this report?**

Our findings highlight racial health disparities that exist in psychological, behavioral, and well-being variables among people with knee osteoarthritis. Specifically, the serial-mediated model explained the process of why Black individuals experience lower health-related quality of life than their White counterparts.

**What are the implications for public health practice?**

Health care providers should be aware of discrepancies in physical activity participation that might affect health-related quality of life among people with knee osteoarthritis. They should also prescribe physical activity as a self-management and lifestyle change for Black patients, along with providing holistic interventions that incorporate depression and lifestyle management.

## Introduction

Knee osteoarthritis (knee OA), which is the most common form of osteoarthritis, is a leading cause of disability (1) and affects an individual’s health-related quality of life (HR-QOL) (2). HR-QOL is a multidimensional construct that assesses the extent to which an individual’s health condition or symptoms may interfere with their daily activities, such as physical functioning, social functioning, and mental health (3). HR-QOL has been proven to be disproportionately lower among Black people compared with their White counterparts across several clinical samples, including people with dementia (4), cancer patients (5,6), and stroke patients (7), and this pattern is consistent among people with knee OA (8–10). Therefore, identifying the important biopsychosocial factors that might explain racial disparities attributed to HR-QOL among knee OA patients is an essential step to redress the observed health disparity.

An established determinant of HR-QOL among people with knee OA is physical activity (11). Physical activity provides a multitude of benefits that promote healthy aging and specifically has been found to improve HR-QOL among people who experience chronic pain (12). Additionally, physical activity contributes to joint health by strengthening muscles around the joint and prevents cartilage deterioration, which helps improve knee mobility and in turn leads to improved physical functionality, both of which are determinants of HR-QOL (3). Because of these benefits, physical activity is often recommended as behavioral treatment of knee OA, as even low-intensity physical activity levels are effective in improving HR-QOL (13). Racial disparities exist in physical activity participation; Black people participate less than their White counterparts, and this disparity extends to people with knee OA (14). Being diagnosed with knee OA brings additional challenges that can cause participation in physical activity to deteriorate, specifically among Black people (15). One of the challenges is the high prevalence of depression among people with knee OA that might exacerbate the barriers to participating in physical activity (16) in the form of loss of interest in activities (17) and reduced energy (18).

Symptoms of depression can also manifest from experiencing pain that may hinder people with knee OA from engaging in their activities of daily living and self-care (19). In addition, emerging findings continue to show that Black Americans report experiencing higher levels of pain and disability compared with their White counterparts (20,21). Despite the evidence emphasizing racial disparities in pain, depression, and physical activity participation, how these factors explain the racial disparities in HR-QOL is unclear. 

The rationale for the proposed conceptual model was derived from the Biopsychosocial Model of Pain (BPS Model) for osteoarthritis pain, where experiencing pain is the core of psychological (stress, mood, depression), behavioral (sleep, exercise, diet), and social (occupation, social support) dysfunction among knee OA patients, which accounts for biological factors (22). Although pain can affect each factor independently, the BPS Model demonstrates a dynamic relationship among psychological, behavioral, social, and biological factors. For instance, experiencing pain contributes to increasing the likelihood of developing affective disorders such as anxiety and depression (23). A negative affect such as depression may be a pre-existing risk factor for chronic pain; however, the result of a causal analysis indicated that pain caused depression among rheumatoid arthritis patients over a 6-month period (24). Recent studies have supported this causal relationship (25), and a systematic synthesis of the top 100 studies that cited pain and depression found that 47% demonstrated a causal relationship between pain and depression (26,27). Furthermore, depression is a psychological factor that has a behavioral impact resulting in less interest in self-care behaviors such as physical activity. In a longitudinal study that tracked women for 32 years, higher depression predicted less subsequent physical activity (28). Physical activity is a prominent determinant of HR-QOL among people with knee OA, as supported by systematic reviews (29,30), as are other factors in the BPS model such as pain (31) and depression (32). To determine whether relationships among the discussed factors from the BPS model are distinct between Black and White people, we developed a conceptual model that proposes the direct and indirect relationships among pain, depression, physical activity, and HR-QOL accounting for race (Figure). We aimed to understand the determinants of HR-QOL disparities associated with knee OA among Black and White people, using a large sample of patients with knee OA.

**Figure:**
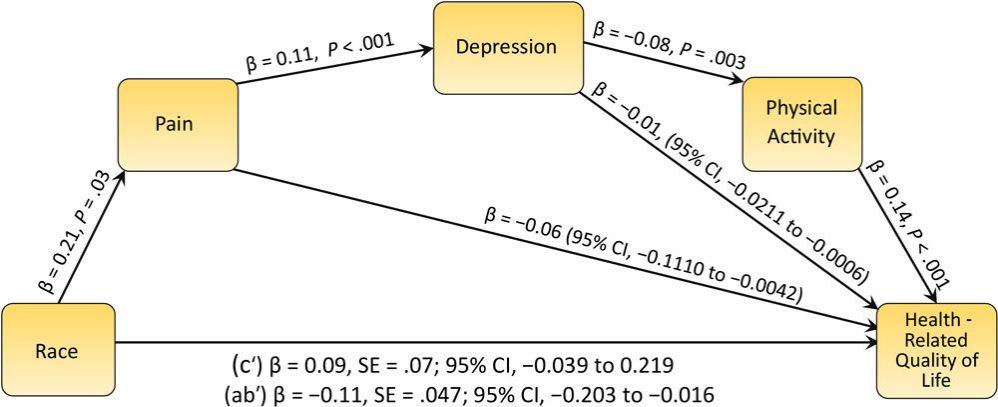
Mediation model of the association between race and quality of life by pain, depression, and physical activity (N = 1,498).

## Methods

The primary objective of this study was to test racial differences in physical activity, HR-QOL, depression, and pain among a large sample of people with knee OA. In accordance with the literature and mentioned postulations, we hypothesized that Black people would report higher levels of pain and symptoms of depression and lower physical activity levels and HR-QOL, compared with their White counterparts. The secondary objective was to test the conceptualized serial mediation model. We hypothesized that race would predict pain (H1), and pain would predict depressive symptoms (H2). Depressive symptoms would predict physical activity (H3), which in turn would predict HR-QOL (H4). We also hypothesized that these pathways would mediate the relationship to explain why Black people with knee OA experience lower HR-QOL (H5).

The Osteoarthritis Initiative is an ongoing observational study funded by the National Institutes of Health that tracks people who are at high risk for or are diagnosed with knee OA at the time of the study. The study was conducted at 4 sites: Baltimore, Maryland; Columbus, Ohio; Pittsburgh, Pennsylvania; and Pawtucket, Rhode Island. The study received institutional review board approval at each participating site and at Northwestern University. We conducted a secondary data analysis using data from 2006 through 2014 from the Osteoarthritis Initiative Data Repository (https://nda.nih.gov/oai/) (33).

Participants (N = 2,603) were adults diagnosed with knee OA who self-identified as White (n = 2,088) or Black (n = 515). All participants provided informed consent before enrollment. In the original study, data were collected on 9 occasions; we used data that were collected at baseline and year 8. 

### Measures

We analyzed data from the latest released variables that were relevant for the investigation, which was identified as meeting/year 8. The following measures collected at baseline (T1) and year 8 (T2) were used for the study.

**Knee pain.** The pain subscale (5 items) of the Western Ontario and McMaster Universities Osteoarthritis Index (WOMAC) is a psychometrically sound instrument that was used to assess the experience of pain symptoms related to knee OA (34).

**Depression symptomology.** Symptoms of depression were measured using the Center for Epidemiologic Studies Depression Scale (CES-D-20) (35). The CES-D-20 is a 20-item questionnaire that assesses 4 domains: positive mood (4 items), depressed mood (8 items), physical symptoms (6 items), and interpersonal relationships (2 items). Each item is framed on a scale of frequency of symptoms that range from 0 (little or no symptoms) to 3 (often). CES-D-20 total score ranges from 0 to 60 with a score of 16 or higher indicating depressive symptoms (35,36). Research suggests that a higher score is correlated with poorer health (35).

**Physical activity.** The Physical Activity Scale for the Elderly (PASE) was administered to measure physical activity levels. PASE comprises 12 items that assess a participant’s activities during the previous week to report the frequency, duration, and intensity (light, moderate, strenuous) of activities such as walking outside of home, strength or endurance exercise, light and heavy housework, lawn work, home repair, gardening, caregiving, sport and recreational activities (with light, moderate, or strenuous effort), and work (for pay or volunteer). Participants were asked, “Over the past 7 days, how often did you do any exercises specifically to increase muscle strength and endurance, such as lifting weights or pushups, etc.?” with responses of 1) never, 2) seldom (1 or 2 d/wk), 3) sometimes (3 or 4 d/wk), and 4) often (5–7 d/wk). Response options rated the frequency of the activities as less than 1 hour per day, 1 to less than 2 hours per day, 2 to 4 hours per day, and more than 4 hours per day. Overall PASE scores can range from 0 to 739, with higher scores indicating higher levels of physical activity. PASE has shown excellent reliability among people with knee pain (37).

**Health-related quality of life.** The Short Form Health Survey 12 (SF-12) was administered to measure HR-QOL (3). The SF-12 assesses multiple domains including general health, physical functioning, mental health, and role limitations that stem from physical and mental problems. Participants were asked to consider their experience over the past 4 weeks to rate items such as “How often did physical health result in being limited in kind of work or other activities?” and “How much did pain interfere with normal work (include work outside home and housework)?” Responses options were 1) not at all, 2) a little bit, 3) moderately, 4) quite a bit, and 5) extremely. Four items of the SF-12 have reversed scoring (items 1, 8, 9, and 10), with a total score range from 0 to 100 where higher scores indicate better HR-QOL (38).

### Analysis

We conducted normality tests based on recommendations and guidelines (39) and reported participant characteristics and measures of central tendency and correlations for variables of interest. The primary hypothesis was tested using 2 sets of analysis of variance (ANOVA) models to assess whether race (Black or White) of participants was associated with differences in pain, depression, physical activity, and HR-QOL at baseline and follow-up. Significant differences in outcome variables were further tested in the mediation analyses to determine whether change in these outcomes was determined by race. The serial mediation model was constructed and tested by using the PROCESS macro (v. 4.1) for SPSS (IBM Corporation) (40). All variables in the model were reported as changes in *z*-score values by regressing the Time-2 variable over its baseline (Time-1) measure, which is a well-documented approach (41,42). Significance was determined by using 95% bias-corrected CIs, which were computed from 5, 000 bootstrapped samples. The model tested whether race predicted change in pain scores (X1), followed by whether pain scores predicted depression (X2), and whether depression predicted change in physical activity (X3). Finally, we tested whether physical activity predicted change in HR-QOL (X4). This pathway represents the serial mediation, and successful mediation was denoted if the CI of the total indirect effect (X1–X4) did not include zero and if the direct pathway was no longer significant (40). The model controlled for nonmutable covariates including socioeconomic status (education, income, occupation) (43) and age. The model was then reanalyzed to additionally control for mutable factors, specifically body mass index (BMI).

## Results

Of the 2,603 participants analyzed, 80.2% (n = 2,088) were White and 19.8% (n = 515) were Black; the average age of White participants was 68.6 (SD, 9.0) years, and the average age of Black participants was 65.9 (SD, 8.4) years (Table 1). The average BMI was 28.9 (SD, 4.6) kg/m^2^ for White participants and 32.2 (SD, 4.8) kg/m^2^ for Black participants; most participants were female (n = 1,516 [58.2%]). Normality tests indicated that baseline values of pain, depression, physical activity, and HR-QOL were within normal range (skewness: 0.59; kurtosis: −0.002).

**Table 1:** Demographic Characteristics of the Sample, Osteoarthritis Initiative, 2006–2014^a^

Variable	White (n = 2,088)	Black (n = 515)	*P* value (statistical test value)^b^
**Age, mean (SD) [range], y**	68.6 (9.0) [51–85]	65.9 (8.4) [50–85]	<.001 (*F = *26.7)
**Body mass index, mean (SD) [range], kg/m^2^**	28.9 (4.6) [16.9–46.8]	32.2 (4.8) [20.3–48.7]	<.001 (*F = *202.0)
**Sex**			
Male	938 (44.9)	149 (28.9)	<.001 (χ^2^ = 43.4)
Female	1,150 (55.1)	366 (71.1)	
**Income, $**			
<10,000	40 (2.0)	55 (12.1)	<.001 (χ^2^ = 207.7)
10,000–24,999	181 (9.2)	89 (19.6)	
25,000–49,999	500 (25.5)	158 (34.7)	
50,000–99,999	754 (38.5)	119 (26.2)	
>100,000	483 (24.7)	34 (7.5)	
**Education**			
High school graduate	258 (12.8)	106 (21.2)	<.001 (χ^2^ = 173.2)
Some college	482 (23.9)	190 (37.9)	
College graduate	477 (23.6)	56 (11.2)	
Some graduate school	191 (9.4)	28 (5.6)	
Graduate degree	612 (30.3)	70 (14.0)	
**Employment**			
Works for pay	1,207 (58.2)	304 (59.7)	<.001 (χ^2^ = 59.9)
Unpaid work for family business	28 (1.3)	8 (1.6)	
Not working for health reasons	79 (3.8)	60 (11.8)	
Not working for other reasons	761 (36.7)	137 (26.9)	

a Values are no. (%) unless otherwise indicated. Values may not sum to total because of missing data.

b One-way analysis of variance was used for continuous variables, and the χ^2^ test was used for categorical variables.

Bivariate correlations indicated that race was correlated with pain (T1: *r* = 0.35, *P* < .001; T2: *r* = 0.28, *P* < .001), depression (T1: *r* = 0.11, *P* < .001; T2: *r* = 0.11, *P* < .001), physical activity (T1: *r* = −0.06, *P* = .002; T2: *r* = 0.09, *P* < .001), and HR-QOL (T1: *r* = −0.27, *P* < .001; T2: *r* = −0.18, *P* < .001). The ANOVA that tested baseline differences between races found significant differences in pain (*F*_1,2579_ = 365.33, *P* < .001), depression (*F*_1,2340 _= 29.30, *P* < .001), physical activity (*F*_1,2587_ = 9.83, *P* = .002), and HR-QOL (*F*_1,2567 _= 205.73, *P* < .001). Black participants experienced higher pain 10.0 (SD, 7.7) and depression 26.5 (SD, 7.4), with poorer HR-QOL 42.4 (SD, 8.7) and lower levels of physical activity 144.8 (SD, 87.0) compared with their White counterparts (Table 2). The same pattern was observed at follow-up: Black participants indicated worse pain 8.9 (SD, 8.4) and depression 26.3 (SD, 6.1) and lower HR-QOL 42.1 (SD, 9.3) and levels of physical activity 128.9 (SD, 82.0) compared with their White counterparts. The significant differences in health determinants between races at both points supported the testing of these variables in the serial mediation model. The remaining correlations can be found in Table 3. The serial mediation model (Figure) indicated that race predicted pain (β = 0.21, *P* = .03), pain to predict depression, (β = 0.11, *P* < .001), depression to predict physical activity, (β = −0.08, *P* = .003), and physical activity to predict HR-QOL (β = 0.14, *P* < .001). The total indirect pathway found race to mediate the relationship from X1 to X4 (β = −0.11, SE = .047; 95% CI, −0.203 to −0.016), in favor of White race of higher HR-QOL, while the direct pathway crossed through zero (β = 0.09, SE = .07; 95% CI, −0.039 to 0.219), thus meeting the requirements for successful mediation effect. The same pattern was found when also controlling for BMI, which yielded a significant indirect effect (β = −0.11, SE = .051; 95% CI, −0.213 to −0.009), and a nonsignificant direct effect (β = 0.12, SE = .07; 95% CI, −0.012 to 0.260). 

**Table 2:** Participant Scores for Outcome Variables at Baseline and Week 96, by Race, Osteoarthritis Initiative, 2006–2014

Outcome variable	White	Black	ANOVA *P* value (*F*)
	Mean (SD) [range]		
**Baseline**			
Pain	4.6 (5.1) [0–33]	10.0 (7.7) [0–39]	<.001 (365.3)
Depression	24.8 (5.3) [20–58]	26.5 (7.4) [20–61]	<.001 (29.3)
Physical activity (PASE)	157.3 (78.8) [0–465]	144.8 (87.0) [0–504]	.002 (9.8)
Quality of life	47.1 (6.0) [16–56]	42.4 (8.7) [14–55]	<.001 (205.7)
**Follow-up**			
Pain	4.5 (5.1) [0–28]	8.9 (8.4) [0–36]	<.001 (167.1)
Depression	25.9 (5.8) [20–64]	26.3 (6.1) [20–68]	<.001 (22.1)
Physical activity (PASE)	148.2 (81.1) [0–570]	128.9 (82.0) [0–429]	<.001 (16.4)
Quality of life	45.7 (7.0) [16–56]	42.1 (9.3) [16–56]	<.001 (67.6)

Abbreviations: ANOVA, analysis of variance; PASE, Physical Activity Scale for the Elderly.

**Table 3:** Zero-Order Correlations Among Study Variables, Osteoarthritis Initiative, 2006–2014^a^

Construct	1.	2.	3.	4.	5.	6.	7.	8.	9.
1. Race									
**Baseline**									
2. Pain	.352^b^	—							
3. Depression	.111^b^	.253^b^	—						
4. Physical Activity	−.062^b^	−.035	−.012	—					
5. Quality of Life	−.272^b^	−.546^b^	−.657^b^	.136^b^	—				
**Follow-up**									
6. Pain	.276^b^	.573^b^	.269^b^	−.009	−.436^b^	—			
7. Depression	.109^b^	.212^b^	.551^b^	−.007	−.493^b^	.280^b^	—		
8. Physical Activity	−.093^b^	−.046^c^	−.040	.515^b^	.139^b^	−.055^c^	−.081^b^	—	
9. Quality of Life	−.182^b^	−.378^b^	−.447^b^	.126^b^	.640^b^	−.508^b^	−.623^b^	.256^b^	—

^a^ Race coded as 1 = White, 2 = Black.

^b^ *P* < .01.

^c^ *P* < .05.

## Discussion

We found that, compared with their White counterparts, Black people experienced higher levels of pain and depressive symptoms and lower physical activity participation and HR-QOL, at baseline and follow-up. The secondary objective, which included testing a set of hypotheses related to the conceptual model, was also supported. The serial mediation model indicated that disparities experienced in pain, depression, and physical activity explained why Black people with knee OA experience lower HR-QOL than White people with knee OA.

We also found that Black participants experienced disparities in biopsychosocial predictors, which included higher levels of pain and symptoms of depression compared with White participants. These findings correspond with previous investigations on racial discrepancies in pain across various clinical groups (44–46), including patients with knee OA (20). Although racial disparities in experiencing depressive symptoms have been noted across community-dwelling older adults (44) and in clinical groups that include cancer (45) and stroke (46) patients, our findings among people with knee OA are congruent with and extend the literature to further highlight the importance of this disparity (47) by showing what factors might contribute to racial disparities attributed to depressive symptoms. Specifically, the predictive pathways among biopsychosocial determinants that included race to predict pain, which in turn predicted depression, explain this effect. Although pain among people with knee OA can be both static and dynamic, the aversive chronic sensation, combined with conscious regulation on limiting one’s activities, can explain how this determinant predicts depression. Racial disparities of pain can be traced from discrepancies on how the pain is managed. In health care settings, Black patients are more likely to be subjected to stereotypes and false beliefs (48) from their primary care providers. A recent meta-analysis identified disparities in the prescription of medications, specifically regarding physicians refuting the pain experience of Black patients (21). Additionally, health care providers spend less time and provide fewer behavioral pain management approaches (49) with Black patients compared with White patients, which could explain why fewer Black people engage in self-management behaviors for pain control and alleviation (50).

Congruent with our hypotheses, physical activity levels were lower among Black participants than among White participants. In addition to the debilitating effects of depressive symptoms that dampen physical activity behavior, Black people experience inequitable societal experiences that discourage participation (51). This can be attributed to their experience on receiving exercise prescription by health care providers. For instance, people with arthritis, who receive advice to perform physical activity, demonstrate greater participation (52). However, compared with their White counterparts, Black patients with arthritis receive less advice on physical activity (53), which might explain the racial discrepancy. In addition to an absence of encouragement, Black people also experience threatening scenarios in American society. For instance, when riding a bicycle, Black people are more likely to be the victim of accidents or ticketed by police, and to be a victim of violent crime when walking or jogging, compared with White people (54). These racial disparities in safety might contribute to a lower tendency to be physically active in outdoor settings that are cost-free, such as walking on sidewalks, biking on streets, and trekking on trails.

Finally, our hypotheses pertaining to HR-QOL were validated by the conceptual model. In addition to the proximal behavioral determinant (physical activity), racial disparities also found in biopsychosocial predictors (pain and depression) explained why Black people experience lower HR-QOL. These variables form as pillars of the multidimensional outcome and signify both the importance and challenge of addressing disparities in determinants for leveling asymmetrical differences in HR-QOL.

### Strengths and limitations

Our study has strengths that advance the current literature along with respective limitations. We proposed a conceptual model that included biopsychosocial predictors and behavioral determinants that explained why Black people with knee OA experience lower HR-QOL compared with White people. To the best of our knowledge, ours is the first study to employ a conceptual model to provide greater insight into racial health disparities among people with knee OA. Hypotheses that are tested using theory or conceptual framework yield more robust findings, as the effect sizes are in scope of other relevant determinants (55). Additionally, the large sample size that includes longitudinal data collected from multiple study sites, which include commonly used validated measures, are all strength markers of the study. Limitations of this study include the absence of behavior-related physical activity determinants that could provide additional insight and opportunity for investigation, and if there were racial disparities among these determinants, such barriers, motivations, and self-efficacy toward participating in physical activity. Additionally, it is important to note that several individual-level factors can affect the model constructs, such as BMI. However, BMI plays a complex role among people with knee OA as it would likely directly predict pain and moderate the effects between pain and depression, and between depression and physical activity. The PROCESS macro currently does not include a model that accounts for these effects. Nonetheless, we recognize the importance of BMI, and future interventions should focus on helping patients with knee OA obtain a healthy BMI level. Future large-scale observational studies are needed to assess physical activity determinants from a behavior theory, such as the Health Belief Model or Social Cognitive Theory.

### Conclusions

The findings highlight racial health disparities that exist in psychological, behavioral, and wellbeing variables among people with knee OA, to the extent to which Black people experienced worse pain, depression, and HR-QOL while they had lower levels of physical activity compared with their White counterparts. This pattern of disparities remained the same throughout the study timeline. The serial mediated effects further explained the process of relationship between races with pain, depression, and physical activity as potential contributors to HR-QOL among patients with knee OA. In conjunction with the literature (47), the present findings help identify patterns of health disparity in pain, depression, and physical activity related to knee OA, that might be associated with previously observed disparity in knee OA treatment. For instance, studies have demonstrated that people with knee OA who are Black are less likely to undergo total knee arthroplasty, which might lead to the presence of health disparities (56).

Because physical activity and exercise programs have been shown to improve pain, depression, and quality of life among knee OA patients (11–13), research is needed to design community-based physical activity programs that account for racial and cultural differences to provide a safe and motivating climate (14). For instance, among Black populations, churches and community centers have been shown to promote physical activity effectively (57). Finally, programs found to be effective for training health care providers and developing community-based physical activity programs to reduce racial disparities should be advocated for policies that promote equitable health in the United States.
